# Oxidative Damage to DNA and Lipids as Biomarkers of Exposure to Air Pollution

**DOI:** 10.1289/ehp.0901725

**Published:** 2010-04-27

**Authors:** Peter Møller, Steffen Loft

**Affiliations:** Department of Public Health, Section of Environment Health, University of Copenhagen, Copenhagen, Denmark

**Keywords:** biomarker, DNA damage, lipid peroxidation products, oxidative stress, particulate matter

## Abstract

**Background:**

Air pollution is thought to exert health effects through oxidative stress, which causes damage to DNA and lipids.

**Objective:**

We determined whether levels of oxidatively damaged DNA and lipid peroxidation products in cells or bodily fluids from humans are useful biomarkers of biologically effective dose in studies of the health effects of exposure to particulate matter (PM) from combustion processes.

**Data sources:**

We identified publications that reported estimated associations between environmental exposure to PM and oxidative damage to DNA and lipids in PubMed and EMBASE. We also identified publications from reference lists and articles cited in the Web of Science.

**Data extraction:**

For each study, we obtained information on the estimated effect size to calculate the standardized mean difference (unitless) and determined the potential for errors in exposure assessment and analysis of each of the biomarkers, for total and stratified formal meta-analyses.

**Data synthesis:**

In the meta-analysis, the standardized mean differences (95% confidence interval) between exposed and unexposed subjects for oxidized DNA and lipids were 0.53 (0.29–0.76) and 0.73 (0.18–1.28) in blood and 0.52 (0.22–0.82) and 0.49 (0.01–0.97) in urine, respectively. The standardized mean difference for oxidized lipids was 0.64 (0.07–1.21) in the airways. Restricting analyses to studies unlikely to have substantial biomarker or exposure measurement error, studies likely to have biomarker and/or exposure error, or studies likely to have both sources of error resulted in standardized mean differences of 0.55 (0.19–0.90), 0.66 (0.37–0.95), and 0.65 (0.34–0.96), respectively.

**Conclusions:**

Exposure to combustion particles is consistenly associated with oxidatively damaged DNA and lipids in humans, suggesting that it is possible to use these measurements as biomarkers of biologically effective dose.

Exposure to particulate matter (PM) from combustion processes contributes substantially to cardiovascular and pulmonary ill health and premature mortality globally (Brook 2008; Salvi and Barnes 2009). PM represents highly complex mixtures with large variations in size, chemical composition, shape, surface, reactivity, and charge, in both time and space, due to variable sources, atmospheric chemical reactions, and meteorological conditions (Sioutas et al. 2005). Nevertheless, for exposure assessment for epidemiological association with health outcomes and regulation, PM is usually only considered as mass defined by size cutoff at 2.5 and 10 μm in aerodynamic diameter (PM_2.5_ and PM_10_, respectively). These measures are often little affected by ultrafine particles (UFPs) from, for example, diesel engine emission because of their low mass, although UFPs are thought to have important health effects due to their high alveolar deposition, small size (≤ 0.1 μm in aerodynamic diameter), large surface area, and potential to translocate (Delfino et al. 2005). Moreover, in epidemiological studies, exposure levels are often assigned crudely and groupwise according to air monitoring data and sources near the residence, because the modest individual risks require large numbers and long observation times to assess. Personal exposure can be assessed by portable monitors or carefully registered time–activity patterns in well-defined microenvironments of exposure in small numbers of subjects (Zou et al. 2009). However, the internal dose of PM also depends on breathing patterns and airway deposition, and cardiovascular and other systemic effects of PM require further translocation of PM constituents or signaling molecules or cells from the airways (Mills et al. 2009). Oxidative stress with inflammation is thought to be central in the mechanisms of action for both the pulmonary and extrapulmonary health effects of PM (Mills et al. 2009; Risom et al. 2005). Thus, biomarkers of oxidative stress should serve as proxy measures of the true internal exposure to PM to compare potential health impacts of different sources in both small controlled exposure settings and large population approaches. Oxidative modification of DNA and lipids are particularly relevant for cancer and cardiovascular disease where oxidative stress in the circulation is important (Mills et al 2009; Risom et al. 2005). Experimental studies in animals and cell cultures have consistently shown that combustion-related PM induces oxidative stress and DNA damage in relevant organs and cells (Møller et al. 2008a, 2010). In our experience the effects on biomarkers of oxidized DNA and lipids are observed within a lag period < 24 hr after the exposure to PM.

A number of studies of PM exposure in humans have applied biomarkers of oxidative damage to DNA and lipids in the blood compartment or in terms of products excreted in urine or exhaled breath condensate (EBC), as outlined in Table 1. The biomarkers of oxidatively damaged DNA include 8-oxo-7,8-dihydroguanine (8-oxoGua) or the corresponding deoxynucleoside 8-oxo-7,8-dihydro-2′-deoxyguanosine (8-oxodG) measured in DNA and urine, the exocyclic M_1_ adduct to guanine (M_1_dG), and lesions detected as sites in DNA sensitive to formamidopyrimidine DNA glycosylase (FPG) and endonuclease III (ENDOIII). The biomarkers of lipid peroxidation (LPO) products include conjugated dienes (CDs), lipid hydroperoxides, malondialdehyde (MDA), thiobarbituric acid–reactive substances (TBARS), and F_2_-isoprostanes measured in EBC, plasma, serum, or urine. However, a systematic approach is required to evaluate the validity of their use as biomarkers of biological effective dose in this context. We undertook a systematic review of the published studies to assess the extent and consistency of associations between exposure to combustion-related PM and the biomarkers of oxidative damage to DNA and lipids.

## Materials and Methods

### Studies included in meta-analysis

The publications were identified by searches in the PubMed, EMBASE, and Web of Science databases, as well as reference lists in the identified papers [see Supplemental Material for the search strategy (doi:10.1289/ehp.0901725)]. We included studies that investigated measures of effects of environmental air pollution exposure. This encompassed studies of subjects who had been exposed at work to environmental air pollution (e.g., policemen exposed to traffic exhaust). We excluded studies on occupational exposures to air pollution, such as metal smelting or incineration, because these are characterized by exposure to other air pollution components than those found in urban air. We searched for publications with reported data on LPO products and oxidatively damaged DNA by means of the biomarkers outlined in Table 1 in airways, the blood compartment, and urine. Isolated leukocytes, lymphocytes, or mononuclear blood cells are all referred to as white blood cells (WBCs). We used the term “oxidized nucleobases” for the urinary excretion of 8-oxodG and 8-oxoGua because they are not measured in DNA. Tables 2–5 summarize details of the included studies. The results from some of the studies have been reported in multiple publications; we discuss these as studies rather than as individual publications because they originated from the same investigation.

We stratified the studies into three broad categories: controlled exposures, panel studies, and cross-sectional studies. Studies with controlled exposure to air pollution PM are the most robust type of design as either crossover studies or parallel groups of subjects exposed to air pollution constituents or filtered air. In panel studies, samples are collected from the same individuals at different times of the year in order to exploit contrasts in exposure due to temporal changes. This study design minimizes the influence of interindividual variation because the subjects are their own controls. However, the design is vulnerable to confounding because other factors such as diet and sunlight show temporal (e.g., seasonal) variation, which can affect the value of the biomarker as shown, for instance, for DNA damage in WBCs detected by the comet assay (Møller and Loft 2006; Møller et al. 2002). In addition, the quality of the panel study depends on the exposure characterization; personal exposure characterization shows a closer association with biomarker levels than does exposure assessed from stationary monitoring stations (Sørensen et al. 2003a, 2003b, 2003c; Vinzents et al. 2005). Cross-sectional studies have a less controlled design than do the panel studies because the exposure gradient is obtained by collecting samples from subjects from different geographical areas or occupations. The cross-sectional studies can have optimal exposure characterization, but confounding can be a problem because individual factors such as lifestyle, including diet, may influence the biomarker and covary with the exposure. This problem typically arises, for instance, when policemen and office personal or subjects from rural and urban areas are being compared.

**Figure 1:**
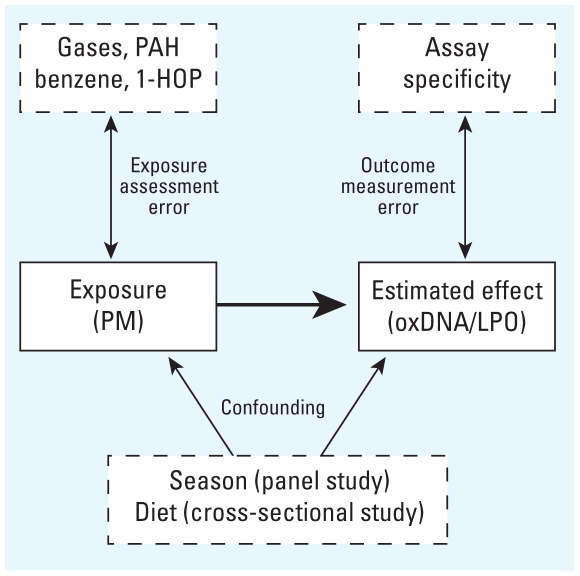
Types of errors in studies of the effect of combustion air pollution. oxDNA, oxidatively damaged DNA.

We critically analyzed the studies with special focus on suboptimal exposure assessment and the quality of biomarkers. We referred to these problems as potential measurement error in the exposure assessment and biomarkers because they can be regarded as systematic errors that may affect the validity. Figure 1 outlines the relationship between the measurement error in exposure assessment and biomarker. We should emphasize that specific studies included in our analysis may have other sources of bias, including selection bias and small numbers of observations, which can affect the estimated effect size in a particular study.

### Measurement error in exposure assessment

The primary exposure assessment in our analysis is the mass concentration of particles as PM_2.5_ or PM_10_ or the number concentration of UFPs. The exposure characterization encompasses data obtained from stationary monitor stations and personal monitors. We regarded personal exposure to PM as the optimal exposure assessment; for studies without PM measurements we regarded data on ambient gasses [nitrogen oxides (NO_x_), ozone (O_3_), or sulfur dioxide], polycyclic aromatic hydrocarbons (PAHs), and benzene as indirect estimates of PM exposure with greater potential for error. Similarly, the urinary excretion of metabolites of PAHs [e.g., 1-hydroxypyrene (1-HOP)] and benzene [*S*-phenylmercapturic acid (*S*-PMA) and *trans*,*trans*-muconic acid (*tt*-MA)] generated by their biotransformation are potentially biased estimates of the ambient concentration of PM, although they may be important biomarkers of internal dose of the parent compound. The measured PM showed the strongest association with the biomarkers of oxidized DNA and lipids in studies that measured the exposure as both PM and nitrogen dioxide (NO_2_), 1-HOP, *S*-PMA, and *tt*-MA (Avogbe et al. 2005; Bräuner et al. 2007; Sørensen et al. 2003a, 2003b). We expect that the measurement error will be nondifferential, which usually biases effect estimates toward the null because they tend to obscure contrasts between the exposed and unexposed and those with or without the outcome of interest.

### Measurement error related to biomarkers

The potential for biomarker measurement error originates from unspecific measurements or analytic flaws due to poor assay conditions. For instance, suboptimal assay procedures used to detect oxidatively damaged DNA may cause spurious oxidation that increases the apparent level of DNA damage. The level of 8-oxodG in DNA from unexposed mammals is approximately 1 lesion/10^6^ dG (deoxyguanosine); the European Standards Committee on Oxidative DNA Damage (2003a, 2003b; Gedik et al. 2005) recommended that publications that report levels of 8-oxodG above a threshold of 5 lesions/10^6^ dG should be interpreted with caution. The comet assay detects DNA damage by migration of DNA in agarose gels, and the end points are usually reported as extent of migration, although they can be transformed to lesions per unaltered nucleotides by calibration with ionizing radiation (Forchhammer et al. 2008; Møller et al. 2004). The level of oxidatively damaged DNA measured by the comet assay in WBCs of humans is < 1 lesion/10^6^ nucleotides (Møller 2006). Oxidatively damaged DNA, nucleobases, and LPO products can be measured by antibody-based methods, but artificially high background levels can occur because of unspecific binding of the antibodies to other biomolecules (Halliwell and Whiteman 2004; Møller et al. 2008b). The simple assay of TBARS and CDs has been seriously criticized and is not recommended for *in vivo* detection of LPO products, whereas improved methods using high-performance liquid chromatography (HPLC) purification steps are more reliable assays of TBARS (Halliwell and Whiteman 2004). We classified biomarkers with suboptimal biochemical analysis as follows: (1) simple spectrophotometric measurement of TBARS without a prepurification step; (2) simple assays for CDs and lipid hydroperoxides, (3) levels of 8-oxodG exceeding a threshold of 5 lesions/10^6^ dG in the unexposed group, and (4) detection of oxidatively damaged DNA, nucleobases, or lipids by antibody-based methods without prepurification steps. We expect that the biomarker measurement error will result in reduced effect estimates for both nonspecific biomarkers and assays having low sensitivity or a high limit of detection.

### Assessment of estimated effect size

The studies differ considerably in design, and the results are reported in ways and units that preclude direct comparison of the estimated effect size in the studies. Thus, we have estimated the effect of exposure on biomarkers as standardized mean differences with 95% confidence intervals (CIs) between exposed subjects and referents in a random effects meta-analysis by means of Review Manager (RevMan; version 5.0; Nordic Cochrane Centre, Cochrane Collaboration 2008, København Ø, Denmark). The standardized mean difference is the difference in means of groups divided by the pooled standard deviation (SD). It is a measurement of estimated effect, which can be used in a meta-analysis when all studies assess the same outcome (level of oxidized biomolecules in this analysis), but it is measured in a variety of ways with different scales. We assessed the estimated effect size in a random model meta-analysis because it incorporates heterogeneity among studies. The heterogeneity between studies was analyzed by tau squared, chi squared, and *I*^2^ tests; tests for subgroup differences were carried out using the chi-square test. We obtained means, SDs, and the number of subjects from the studies, or we calculated these values from data reported in the publications (see Tables 2–5). The variance was reported in different ways in the original publications; thus, our estimates of 95% CIs may be biased, but the central estimates (means) should not be. We calculated the mean and SD from regression analyses for studies that modeled continuous data and defined the exposure gradient as equal to either the interquartile range (Kelishadi et al. 2009; Liu et al. 2009a, 2009b) or a 10 μg/m^3^ increase in PM (Liu et al. 2007). The interquartile range in PM_2.5_ was in the same range as a 10-μg/m^3^ increase in PM_2.5_ in the studies carried out in Windsor, Ontario, Canada (Liu et al. 2007, 2009a, 2009b), whereas the interquartile range (66.5 μg/m^3^ measured as PM_10_) measured by Kelishadi et al. (2009) in Iran was substantially higher, which could reflect different sources of exposure or PM fraction. We calculated overall means and SD for studies that included more than one group of exposed subjects or investigated the same subjects under different exposure scenarios (Avogbe et al. 2005; De Coster et al. 2008; Mills et al. 2008; Novotna et al. 2007; Rundell et al. 2008; Singh et al. 2007; Staessen et al. 2001; Sørensen et al. 2003a, 2003b). We assumed that the interquartile ranges would be equal to the SDs found in studies that used nonparametric analyses and reported variation as ranges (Chen et al. 2007; Romieu et al. 2008; Sørensen et al. 2003a, 2003b).

## Results

### Oxidative damage reported in controlled exposure studies

Table 2 summarizes studies on the association between air pollution exposure and oxidized DNA and lipids in controlled exposure studies. The number of subjects in these studies is rather low (3–41 subjects; mean ± SD, 18 ± 12), which may be because carrying out controlled exposure on a large number of subjects is demanding. A study of air pollution exposure to UFPs in persons bicycling for approximately 90 min in a laboratory or on traffic-intense streets reported that the level of FPG sites in WBCs was associated with the number concentration of UFPs (Vinzents et al. 2005). A subsequent investigation by the same group had a similar correlation between particulate fractions with median particle sizes of 23 nm and 57 nm (consistent with semivolatile organic compounds from diesel exhaust and carbonaceous soot emissions into the air of a busy street) and the level of FPG sites in WBCs (Bräuner et al. 2007). A subsequent study in elderly subjects showed a statistically non-significant decrease in urinary excretion of the F_2_-isoprostane 8-iso-prostaglandin-F_2_ after a period of home air filtration (Bräuner et al. 2008). In another study of elderly patients with coronary heart disease, Mills et al. (2008) reported that inhalation of concentrated air pollution particles (CAPs) was associated with increased concentration of 8-isoprostanes in EBC. This finding is in keeping with that found by Rundell et al. 2008) who observed that healthy young subjects had elevated level of MDA in EBC after intensive exercise at a location with high-traffic intensity compared with the same type of exercise at a location with less traffic.

Controlled exposure to wood smoke containing very high mass concentration of particles has been associated with increased levels of LPO products in serum, urine, and EBC (Barregard et al. 2006, 2008). However, Danielsen et al. (2008) observed no association between exposure and FPG sites in WBCs and suggested that this result may be due to increased DNA repair activity of oxidized nucleobases because urinary excretion of 8-oxoGua and WBC expression levels of the 8-oxoguanine DNA glycosylase (*OGG1)* base excision repair enzyme, which removes 8-oxoGua from DNA, were increased after exposure to wood smoke but not after exposure of the same subjects to clean air. Increased urinary excretion of 8-oxoGua was also observed in a study where subjects were exposed to exhaust on a traffic-intense street for 4 hr (Suzuki et al. 1995), but 2 hr of exposure to a high concentration of diesel exhaust was not associated with urinary excretion of 8-oxodG or F_2_-isoprostanes in subjects with metabolic syndrome (Allen et al. 2009).

### Oxidative damage reported in panel studies

Table 3 summarizes studies on the association between air pollution exposure and oxidized DNA and lipids in panel studies. These studies involved multiple measurements over time, and the number of subjects in these studies was higher than the number of subjects in controlled exposure studies (2–182 subjects; mean ± SD, 62 ± 59). Several panel studies showed that concurrent air pollution exposure was associated with elevated levels of LPO products in EBC (Liu et al. 2009a; Romieu et al. 2008) and plasma (Liu et al. 2007; Medina-Navarro et al. 1997), as well as elevated levels of 8-oxodG in plasma (Chuang et al. 2007). Subjects without doctor-diagnosed cardiovascular diseases or who did not take medication for diabetes showed an association between outdoor levels of PM_2.5_ and TBARS in plasma, although the analysis of all subjects in the study only indicated statistically nonsignificant associations between personal exposure to PM_2.5_ and TBARS or 8-isoprostanes in plasma (Liu et al. 2009b). Personal exposure to PM_2.5_ was associated with increased levels of 8-oxodG in WBCs of students living in the center of Copenhagen, whereas the exposure was only associated with the MDA levels of women and not with the level of FPG sites in WBCs in any group (Sørensen et al. 2003a, 2003b). Interestingly, Sørensen et al. (2003a, 2003b) observed no correlation between 8-oxodG in WBCs and the background mass concentration of PM_2.5_ measured at stationary monitoring stations, suggesting that a relatively clean urban air may provide too little contrast in the long-range transported fractions of PM to be a reliable indicator of traffic-generated exposure. Moreover, they found significant association between the biomarkers and personal exposure to NO_2_ supporting the key role of PM. This is in keeping with observations from a controlled exposure study with constant NO_2_ exposure, which showed a strong effect of change in PM exposure on DNA oxidation (Bräuner et al. 2007).

### Oxidative damage reported in cross-sectional studies

The design of the cross-sectional studies can be grouped into two main categories. The first category is characterized by studies that achieved the exposure contrast by studying subjects in occupations with different ambient air pollution levels (Table 4). The other category is characterized by studies of subjects, often with comparable occupations or ages, from geographical areas with different ambient air pollution levels (Table 5). The cross-sectional studies have generally included more subjects than the controlled exposure studies and panel studies. The number of subjects in the cross-sectional studies on different occupations has been in the range of 31–356 subjects (mean ± SD = 109 ± 95 subjects), whereas the studies that have contrasted exposure in different geographical areas have used even higher number of subjects (43–894 subjects; mean ± SD = 222 ± 234).

Using job titles as the basis for stratification of exposure, studies showed that subjects in occupations with high exposure to traffic emissions had higher levels of FPG sites (Avogbe et al. 2005; Cavallo et al. 2006; Palli et al. 2009) and 8-oxodG and M_1_dG (Ayi Fanou et al. 2006; Singh et al. 2007) in WBCs than did referents. However, the latter two studies reported levels of 8-oxodG that were above the threshold of 5 lesions/10^6^ dG, suggesting the potential for spurious oxidation of the samples. Another study showed higher levels of FPG and ENDOIII sites in WBCs of traffic emission exposed policemen compared with other policemen working indoor during a month when air pollution was relatively high (i.e., January) but no association during a month with low air pollution exposure (i.e., September) (Novotna et al. 2007). In contrast, Bonina et al. (2008) found no difference in serum lipid hydroperoxides concentration when comparing traffic officers with healthy indoor workers; the primary purpose of their study appears to have been to compare lipid hydroperoxide levels between subjects that received phytochemicals and subjects that did not, and they evaluated associations with air pollution in a secondary analysis. Evidence of null associations in studies that evaluate air pollution as a secondary exposure suggests the possibly of a general trend toward publication bias favoring studies that report positive associations when air pollution is the primary exposure of interest, but the findings of the Bonina et al. (2008) study may also have been due to the use of a nonspecific spectro-photometric assay for the detection of LPO products, which can bias the estimated effect toward null. Studies using urinary biomarkers have also shown increased levels of 8-oxodG and 15-F_2t_-isoprostanes in subjects exposed to high concentrations of traffic-vehicle exhausts (Chuang et al. 2003; Lai et al. 2005; Rossner et al. 2008a, 2008b).

Cross-sectional studies of subjects living, working, or going to school in locations with different ambient air pollution levels encompass investigations of subjects with predefined age groups, such as children, adolescents, adults, or elderly. Studies of children living in areas with different levels of exposure have shown positive associations with 8-oxodG levels in nasal cells (Calderón-Garcidueñas et al. 1999), 8-oxodG in WBCs (Buthbumrung et al. 2008), LPO products in plasma (Kelishadi et al. 2009; Vujovic et al. 2009), and urinary excretion of 8-oxodG (Svecova et al. 2009). Studies of air pollution exposure in adults have provided more mixed results: benzene as a marker of urban air pollution exposure was associated with urinary excretion of *S*-PMA and 8-oxodG in WBCs, but not with ENDOIII and FPG sites in WBCs or urinary excretion of 8-oxodG (Sørensen et al. 2003c). Other studies of urinary excretion of 8-oxodG have shown positive associations (De Coster et al. 2008; Loft et al. 1999; Staessen et al. 2001; Wei et al. 2009), but studies of LPO products in plasma have indicated both positive associations with oxidative damage (Chen et al. 2007; Isik et al. 2005; Sanchez-Rodriguez et al. 2005) and no apparent effects (Autrup et al. 1999; Hicks et al. 1996).

### Combined effect estimates for markers in airways, blood, and urine

**Figure 2:**
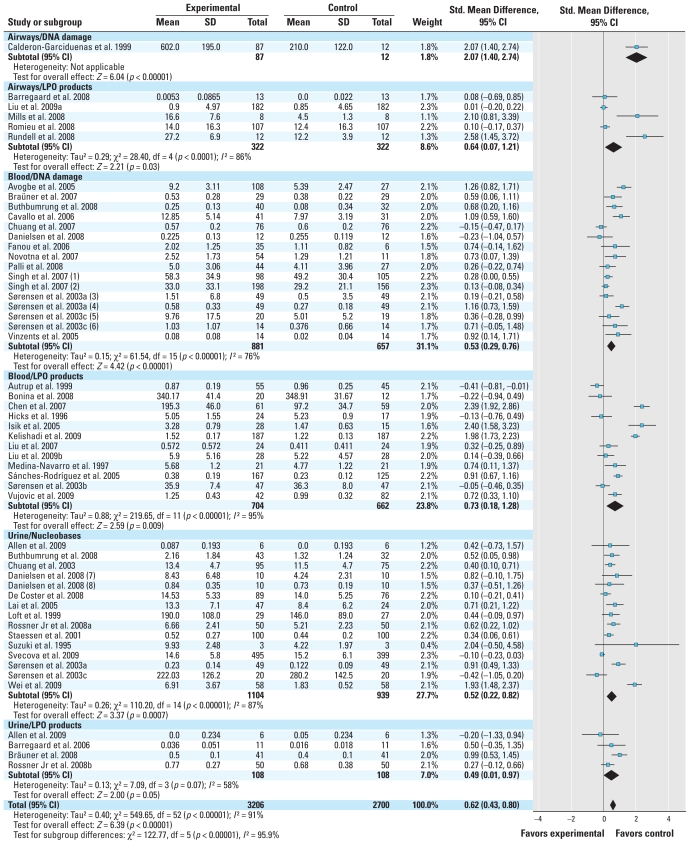
Forest plot of air pollution exposure on biomarkers of oxidized DNA, nucleobases, and lipids. Specific biomarkers in studies that have measured multiple assays of oxidized DNA and lipids are (1) 8-oxodG, (2) M1dG, (3) 8-oxodG, (4) ENDOIII/FPG sites, (5) ENDOIII/FPG sites, (6) 8-oxodG, (7) 8-oxodG, and (8) 8-oxoGua (the numbers in parentheses refer to references citations that are listed by first author/year only).

The qualitative assessment in the preceding sections indicates that most of the reports showed associations between air pollution exposure and oxidatively damaged DNA, nucleobases, and lipids. Most studies measured the biomarkers in surrogate tissue cells such as WBCs or noncellular bodily fluids such as plasma, urine, and EBC. The data on biomarkers of the airways mainly encompass measurements of LPO products in EBC, whereas only one study examined 8-oxodG in nasal cells (Calderón-Garcidueñas et al. 1999). Figure 2 shows study-specific and overall estimates of effect for exposure to PM on oxidatively damaged DNA, nucleobases, and lipids in the airways, blood, and urine. In general there is considerable heterogeneity between the studies and between subgroups; methodological diversity between studies may explain the heterogeneity. In addition, the categorization of the exposure does not take into account that exposure gradients most likely differ between the studies. It is not possible to calculate a standardized exposure unit (e.g., effect per 10-μg/m^3^ increase PM_2.5_) because the studies reported exposure measurements in different fractions of PM, or they contained no information about the concentration of PM. The overall standardized mean differences between exposed subjects and nonexposed referents for the oxidized DNA and for LPO products in the blood were 0.53 (95% CI, 0.29–0.76) and 0.73 (95% CI, 0.18–1.28), respectively. In the urine the estimated effect size by PM exposure versus nonexposed referents for oxidatively damaged DNA and nucleobases and for LPO products was 0.52 (95% CI, 0.22–0.82) and 0.49 (95% CI, 0.01–0.97), respectively. This suggests that exposure to PM is associated with comparable increases in oxidized DNA and lipids, although it should be emphasized that the heterogeneity between subgroups might mask real differences between the biomarkers. The effect on DNA damage in the airways is presently difficult to assess because there was only one study of oxidized DNA (Calderón-Garcidueñas et al. 1999). The estimated effect on LPO products in EBC was 0.64 (95% CI, 0.07–1.21), which is comparable to overall standardized mean differences in LPO products in the blood and urine with PM exposure. This finding suggests that LPO products in plasma and urine are suitable biomarkers of biologically effective PM dose reflecting oxidative stress in the airways.

### Combined estimates according to the potential for exposure or outcome measurement error

The focus of our analysis was on the estimated effect of exposure to PM on biomarkers of oxidized DNA, nucleobases, and lipids. A number of studies have not measured personal exposure to PM, which may be because of their focus on other air pollution constituents or lack of resources or because the study was too large to do personal exposure measurements. Ideally, all studies should have included personal measurements of PM, and the analysis should have included mutual adjustment for coexposures to other air pollutants. We assumed that PM is the most important contributor to oxidative stress among the air pollutants and that personal exposure can be estimated from the personal exposure to the other pollutants or ambient PM levels, although with potential bias. Of the studies identified in our meta-analysis, only Chen et al. (2007) has estimated personal exposure levels using mathematical modeling of data from stationary monitoring stations. We have categorized the studies according to whether or not they characterized exposure using personal versus ambient PM measurements. Based on the categorization of the studies according to the potential measurement error in Tables 2–5, the estimated effect size is 0.55 (95% CI, 0.19–0.90), 0.66 (95% CI, 0.37–0.95), and 0.65 (95% CI, 0.34–0.96) for studies categorized as having no potential measurement error, potential measurement error in either biomarker analysis or exposure assessment, and potential measurement error in both biomarker analysis or exposure assessment, respectively [see Supplemental Material for the Forest plots (doi:10.1289/ehp.0901725)]. The effect size is essentially identical in the three groups, which could be caused by opposite acting effects of potential measurement errors (regression toward null effect) and uncontrolled confounding factors (increased effect size) in panel and cross-sectional studies. The percentages of studies that did not use personal measurements to assess PM exposure were 8% (1 of 12), 36% (4 of 11), and 100% (29 of 29) in the studies categorized as controlled exposures, panel studies, and cross-sectional studies, respectively (χ^2^ = 36.7, *p* < 0.001). The use of error-prone biomarker assays was less likely in the controlled exposure studies (31%; 4 of 13) than in the panel studies (64%; 7 of 11) and cross-sectional studies (59%; 17 of 29), although no differences were found between the distributions (χ^2^ = 3.4, *p* > 0.05). Overall, the quality of the studies, in terms of the likelihood of exposure and outcome measurement error, appears highest in the controlled studies and lowest in the cross-sectional studies. Both of these measurement errors may bias the effect estimate toward null, whereas uncontrolled confounding factors in panel studies and cross-sectional studies most likely increased the estimated effect size. In addition, we emphasize that the studies that measured personal PM exposure and used more accurate biomarker assays mainly investigated the effect of air pollution particles in realistic urban air concentrations (Bräuner et al. 2007, 2008; Rundell et al. 2008; Sørensen et al. 2003a, 2003b; Vinzents et al. 2005), although one study used high concentrations of wood smoke particles (Barregard et al. 2006, 2008; Danielsen et al. 2008). Collectively, associations between PM exposure and biomarkers of oxidative stress estimated by studies likely to have more accurate exposure and outcome measurements cannot be explained by exposure to excessive concentrations of PM. Yet, the controlled exposure studies in our meta-analysis had few numbers of observations, which might reduce the precision with which effects have been estimated. In addition, controlled exposure studies may be more prone to selection bias due to random errors in selection and have limited generalizability because they are restricted to participants with specific characteristics.

## Discussion

Our analysis shows that exposure to combustion particles is consistently associated with elevated levels of oxidatively damaged DNA and nucleobases and LPO products in human blood cells, plasma, urine, and EBC. The association is seen across studies with optimum designs, including controlled or personal exposure assessment and biomarkers with low potential measurement error due to indirect exposure assessment and/or use of biomarkers prone to artifacts. Still, we emphasize that the identified studies are inhomogeneous in design and quality of biomarkers, which weakens our conclusions about specific exposure–effect relationships for particulate air pollution. In addition, few numbers of subjects, especially in the controlled exposure studies, is a minor limitation that might affect the generalizability of our meta-analysis results.

Our critical analysis indicates that the range in exposure to realistic ambient concentrations of combustion particles is associated with a 50% increase in the level of oxidatively damaged DNA, nucleobases, and lipids, supporting the notion that they are suitable biomarkers of the biologically effective dose of PM. However, we caution against the use of suboptimal biomarkers; ideal biomarkers of oxidative damage should detect a major part of the total ongoing oxidative damage *in vivo*, have small assay variation, have smaller intraindividual than interindividual variation, not be confounded by diet, be stable on storage, and have the same level obtained in target and surrogate tissue (Halliwell and Whiteman 2004). They should also have predictive value of risk of disease, which can be firmly assessed only in prospective studies (Loft and Møller 2006). The biomarkers of urinary excretion of 8-oxodG and TBARS in plasma are among the few biomarkers that have been studied in prospective cohort studies; they have predictive value regarding development of lung cancer and cardiovascular diseases, respectively (Loft et al. 2006; Walter et al. 2004). Further development of oxidized DNA and LPO products as biomarkers of biological effective dose of air pollution exposure should focus on the most reliable and well-validated assays, including assays for the measurement of isoprostanes and techniques that consistently measure low background levels of oxidatively damaged DNA and nucleobases. The relevant biomarkers with low potential of measurement error are constantly developed to increase assay capacity toward high throughput, for instance, the comet assay and urinary excretion of 8-oxodG (Henriksen and Poulsen 2009; Stang and Witte 2009). This development will allow the use of these biomarkers of exposure to PM in large-scale population studies.

## Figures and Tables

**Table 1:** Summary of biomarkers of oxidatively damaged DNA, nucleobases, and LPO products used in studies of the effect of combustion particles.

Biomolecule	Description	Assays
Oxidatively damaged DNA or nucleobases		
ENDOIII/FPG	DNA base lesions detected by bacterial ENDOIII or FPG enzymes, representing mainly oxidized purine (including 8-oxodG) and pyrimidine lesions, respectively	Comet assay
8-oxodG	Major oxidation product in nuclear DNA; detection of 8-oxodG in urine or plasma mainly originates from oxidation of deoxyguanosine triphosphate in the nucleotide pool	HPLC-ECD, LC-MS/MS, antibodies
8-oxoGua	Major oxidation product in nuclear DNA; detection of 8-oxoGua in urine or plasma is likely to arise from cleavage of the oxidized base from DNA by repair enzymes (e.g., OGG1)	HPLC-ECD, LC-MS/MS, antibodies
M_1_dG	Exocyclic DNA damage formed by reactive carbonyl compounds released from oxidized lipids	LC-MS, antibodies

LPO products		
CDs	Breakdown products of fatty acids considered to represent an early stage of the LPO process	Spectrophotometry
Lipid hydroperoxides	Reaction product between O_2_ and carbon radical in lipids	Spectrophotometry
MDA/TBARS	Breakdown carbonyl product of LPO; the reaction with thiobarbituric acid forms adducts that can be detected by spectrophotometry; prepurification of urine or plasma before the reaction with thiobarbituric acid can be considered as a specific measurement of LPO products, whereas the simple TBARS assay is highly unspecific	Spectrophotometry
F_2_-isoprostanes	Products that arise mainly from oxidation of arachidonic acid in phospholipids, often referred to as 8-iso-PGF_2α_ or 15-F_2t_-isoprostanes	GC-MS, LC-MS/MS, antibodies

Abbreviations: 8-oxodG, 8-oxo-7,8-dihydro-2′-deoxyguanosine; 8-oxoGua, 8-oxo-7,8-dihydroguanine; CDs, conjugated dienes; ECD, electrochemical detection; ENDOIII, endonuclease III; FPG, formamidopyrimidine DNA glycosylase; GC-MS, gas chromatography–mass spectrometry; HPLC, high-performance liquid chromatography; LC-MS, liquid chromatography–mass spectrometry; LC-MS/MS, liquid chromatography with tandem mass spectrometry; LPO, lipid peroxidation; M_1_dG, exocyclic M_1_ adduct to guanine; MDA, malondialdehyde; *OGG1*, 8-oxoguanine DNA glycosylase; TBARS, thiobarbituric acid–reactive substances. For descriptions and critical assessments of the assays as biomarkers, see Griffiths et al. (2002) and Halliwell and Whiteman (2004).

**Table 2:** Summary of controlled exposure studies on exposure to air pollution PM from combustion processes.

Biomarker	Subjects (*n*)	Sex, age, smoking	Exposure assessment^a^	Potential measurement error	Findings	Study
8-oxodG (ELISA) F_2_-isoprostanes (LC-MS/MS)	Subjects with metabolic syndrome exposed to diesel exhaust or FA for 2 hr (10)	MF 18–49 years NS	PM_2.5_, 4.8 and 205 μg/m^3^ NO, 38.6 and 1,516 ppb NO_2_, 15.5 and 25.5 ppb	Biomarker (8-oxodG)	No difference in urinary excretion	Allen et al. 2009^b^
8-iso-PGF_2_ (ELISA) 8-oxodG, 8-oxoGua (HPLC/GC-MS) FPG sites (comet) MDA (HPLC-FD)	Healthy subjects exposed to wood smoke in a chamber for 4 hr (13)	MF 20–56 years NS	PM_2.5_, 261 and 14–27 μg/m^3^ UFPs, 137,500 and 5,950 particles/cm^3^ NO_2_, 10 and 8.5 ppb	Biomarker (8-iso-PGF_2_ )	Increased urinary excretion of 8-iso-PGF and MDA levels in EBC; unaltered FPG sites (WBCs) and 8-oxodG and 8-oxoGua (urine)	Barregard et al. 2006, 2008; Danielsen et al. 2008^c^
FPG sites (comet)	Healthy subjects exposed in a chamber for 24 hr (29)	MF 20–40 years NS	Personal UFPs, 6,169–15,362 particles/cm^3^ (non-FA), and 91–542 particles/cm^3^ (FA) NO_x_, 25.3 ppb (non-FA), 28.3 ppb (FA), 11.6 ppb (back ground), and 59.5 ppb (busy street) O_3_, 12.1 ppb (non-FA), 4.3 ppb (FA), 30.1 ppb (back ground), and 19.5 ppb (busy street)	No	Decreased levels in WBCs by exposure to FA	Bräuner et al. 2007
8-iso-PGF_2_ (ELISA)	Elderly subjects exposed in the homes (41)	MF 60–75 years NS	Personal UFPs, 10,016 particles/cm^3^ (non-FA) and 3,206 particles/cm^3^ (FA) PM_2.5_, 12.6 (non-FA) and 4.7 μg/m^3^ (FA) NO_2_, 20.0 (non-FA) and 20.0 μg/m^3^ (FA)	Biomarker	Unaltered urinary excretion	Bräuner et al. 2008
8-Isoprostane (ELISA)	Subjects with stable coronary heart disease (12) and controls (12) exposed to CAPs for 2 hr	M 54 ± 2 and 59 ± 2 years NS	UFPs, 99,400 and zero particles/cm^3^ NO_x_, 7.2 and 6.3 ppb SO_2_, 0.13 and 0.13 ppb O_3_, 5.0 and 6.0 ppb	Biomarker	Increased in EBC by CAPs exposure	Mills et al. 2008
MDA (HPLC)	Subjects exercising in location with low and high traffic intensity (12)	M 21± 2 years NS	Personal UFPs, 252,290 and 7,382 particles/cm^3^	No	Increased after exercise at location with high exposure	Rundell et al. 2008^d^
8-oxoGua (HPLC-ECD)	Healthy subjects exposed to traffic exhaust at a street intersection for 4 hr (3)	M 22–25 years NS	None (49,000 cars/12 hr)	Exposure	Increased urinary excretion during the first 12 hr and 24 hr after exposure; normalized levels at 36 and 48 hr after the exposure	Suzuki et al. 1995^e^
FPG sites (comet)	Subjects bicycling in Copenhagen (15)	MF 25 ± 3 years NS	Personal UFPs (32,400 and 13,400 particles/cm^3^) PM_10_, 23.5 μg/m^3^ (street) and 16.9 μg/m^3^ (background) NO_2_, 32.1 and 24.2 μg/m^3^ (street) and 11.3 μg/m^3^ (background)	No	Increased after cycling in the traffic compared with cycling in the laboratory	Vinzents et al. 2005

Abbreviations: ECD, electrochemical detection; ELISA, enzyme-linked immunosorbent assay; FA, filtered air; FD, fluorescence detection; GC-MS, gas chromatography–mass spectrometry; LC-MS, liquid chromatography–mass spectrometry; LC-MS/MS, liquid chromatography with tandem mass spectrometry; M, male; MF, male and female; NO, nitric oxide; NS, nonsmoker; iso-PGF_2_, 8-iso-PGF_2_, 8-iso-prostaglandin F_2_; SO_2_, sulfur dioxide.

a The values represent exposure assessment in the high-exposure and low-exposure group, respectively, unless stated otherwise by specific footnotes.

b We calculated the mean and SD from the mean difference and 95% CI assuming no missing data in the pair analysis.

c We calculated the net difference in MDA from preexposure values and baseline-adjusted the data according to the level of MDA in the group of subjects exposed to filtered air. The SD was calculated from 90% CI.

d We used the mean level of MDA from the exercises at the locations with low and high PM concentration.

e The data correspond to the mean of the whole exposure period (0–48 hr).

**Table 3:** Summary of panel studies on exposure to air pollution PM from combustion processes.

Biomarker	Subjects (*n*)	Sex, age, smoking	Exposure assessment^a^	Potential measurement error	Findings	Study
8-oxodG (ELISA)	Students followed for 3 months (76)	MF 18–25 years NS	PM_2.5_, 12.7–59.5 μg/m^3^ PM_10_, 22.2–87.2 μg/m^3^ SO_2_, 2.8–39.4 ppb NO_2_, 2.8–33.3 ppb O_3_, 22.5–48.3 ppb (stationary monitoring stations)	Biomarker exposure	Positive association between 8-oxodG in plasma and SO_2_ and O_3_; no association with PM_2.5_, PM_10_, and NO_2_	Chuang et al. 2007
TBARS (FD)	Subjects with diabetes mellitus (25) followed for 7 weeks	MF 28–63 years NS	Personal PM_10_, 25.5 (9.8–133) μg/m^3^	Biomarker	Positive association between PM_10_ levels and TBARS in plasma	Liu et al. 2007^b^
TBARS (FD) 8-Isoprostanes (immunoassay)	Asthmatics (182) followed for 4 weeks	MF 9–14 years NS	PM_2.5_, 2.7–14.3 μg/m^3^ SO_2_, 1.3–13.8 ppb NO_2_, 12.3–27.0 ppb O_3_, 7.5–21.0 ppm Stationary monitoring	Biomarker exposure	Positive association between TBARS in EBC and SO_2_, NO_2_ and PM_2.5_, but not with O_3_; the concentration of 8-isoprostanes in EBC was only associated with SO_2_ concentration	Liu et al. 2009a^c^
TBARS (SPM) 8-Isoprostanes (ELISA)	Normal subjects living in Windsor, Ontario, Canada (29) followed for maximally 50 days	MF 64–96 years NS	PM_2.5_, 6.3 (0.9–16.6) μg/m^3^ (personal exposure) and 15.3 (10.4–24.2) μg/m^3^ (outdoor)	Biomarker	No association with personal PM_2.5_ exposure and LPO products in plasma; an association with outdoor PM_2.5_ and TBARS in a subset of subjects without doctor-diagnosed cardiovascular disease or who did not take diabetic medication	Liu et al. 2009b^d^
TBARS (SPM) CDs (SPM)	Medical doctors investigated 1 or 16 weeks after arrival in Mexico City (21)	NR 27–32 years NS	O_3_, 141 ppb (no report of the O_3_ level in original residence)	Biomarker exposure	Increased TBARS in serum after the first week of the stay, normalized in samples obtained 16 weeks after	Medina-Navarro et al. 1997^e^
MDA (FD)	Asthmatics (107) followed for 2–16 weeks (average 8 weeks)	MF 10 ± 2 years NS	PM_2.5_, 27.4 (4.2–89.5) μg/m^3^ NO_2_, 35.3 (13.9–73.5) ppb O_3_, 31.1 (9.8–60.7) ppb (stationary monitoring stations)	Biomarker exposure	Positive association between ambient PM_2.5_ levels and MDA in EBC	Romieu et al. 2008^f^
8-oxodG (HPLC-ECD) FPG sites (comet) MDA (HPLC)	Students living in Copenhagen, Denmark (50) followed for 1 year	MF 20–33 years NS	Personal PM_2.5_, 16.1 (10–24.5) μg/m^3^ PM_2.5_, 9.2 (5.3–14.8 μg/m^3^ (stationary monitoring stations)	No	Correlation between personal exposure to PM_2.5_ and 8-oxodG in WBCs and MDA in plasma (only women); no correlation between PM_2.5_ and FPG sites in WBCs or 24-hr urinary excretion of 8-oxodG; no correlation between biomarkers and stationary (urban background) measurements of PM_2.5_	Sørensen et al. 2003a, 2003b^g^
8-oxodG (ELISA)	Security guards analyzed before and after a work shift (2) followed for 2 months	NR 18–20 years NS	PM_2.5_, 243 (199–460) μg/m^3^	Biomarker	Increased in urine after the work shift	Wei et al. 2009^h^

Abbreviations: ECD, electrochemical detection; ELISA, enzyme-linked immunosorbent assay; FD, fluorescence detection; M, male; MF, male and female; NR, not reported; NS, nonsmoker; SO_2_, sulfur dioxide; SPM, spectrophotometry.

a The values represent exposure assessment in the high-exposure and low-exposure group, respectively, unless stated otherwise by specific footnotes.

b We calculated means and SD from the regression analysis in the study, based on 10-μg/m^3^ increase in PM_10_ and a coefficient of variation of 100%.

c We calculated the mean level of LPO from TBARS and 8-isoprostanes, and the SD from the lower 95% CI assuming that it is similar to the 5th percentile and the coefficient of variation is the same in the exposed and reference group.

d We calculated data from the regression model reported in the original publication. The data correspond to the difference in LPO products from the interquartile range in personal PM_2.5_ exposure. We calculated the SD from the coefficient of variation of data reported as 5th and 95th percentile, assuming that it is equivalent to 95% CI, and the mean level of LPO products from data of TBARS and 8-isoprostanes.

e We calculated the mean level of LPO products from TBARS and CDs.

f We calculated data (means) by regression analysis assuming that the SD is the same as the interquartile range.

g We assumed that the SD and interquartile is the same value for the analysis of 8-oxodG and calculated the mean level of ENDOIII/FPG sites.

h The study encompassed samples from two subjects analyzed on 29 working days.

**Table 4:** Summary of cross-sectional studies on exposure to air pollution PM from combustion processes in humans in different occupations.

Biomarker	Subjects (*n*)	Sex, age, smoking	Exposure assessment^a^	Potential measurement error	Findings	Study
8-oxodG (HPLC-ECD) MDA (HPLC)	Bus drivers (107)	MF 27–60 years NS	1-HOP (urine)	Exposure	Bus drivers in the city center had higher levels of urinary 8-oxodG excretion than did bus drivers from the rural/suburban area; no clear differences between urinary excretions on working days and on days off observed; unaltered MDA in plasma between bus drivers in the city center and rural/suburban area	Autrup et al. 1999, Loft et al. 1999^b^
Lipid hydroperoxides (SPM)	Traffic officers and controls (32)	M 38–52 years S/NS	None	Biomarker exposure	No difference between exposed and controls	Bonina et al. 2008^c^
FPG sites (comet)	Airport personnel (41) and controls (31)	NR 43 ± 9 years S/NS	Stationary sampling of PAHs Urinary 1-HOP (urine)	Exposure	Higher level in WBCs of exposed subjects	Cavallo et al. 2006^d^
8-oxodG (ELISA)	Taxi drivers (95) and controls (75)	M 40 ± 4 and 44 ± 7 years S/NS	1-HOP (urine)	Biomarker exposure	Highest level in urine of exposed subjects	Chuang et al. 2003
8-oxodG (HPLC-ECD)	Taxi-motor drivers and rural controls (41)	M 36 ± 5 years NS	Ambient (stationary) concentration of PAHs and benzene Urinary excretion of *S*-PMA and 1-HOP	Biomarker exposure	Highest level in WBCs of exposed subjects (high background level of 8-oxodG, 11.1 lesions/10^6^ dG)	Ayi Fanou et al. 2006
8-oxodG (ELISA)	Highway toll workers and controls (74)	F 26 ± 6 and 27 ± 5 years S/NS	Traffic intensity and urinary 1-HOP glucuronide excretion	Biomarker exposure	Highest level in urine of exposed subjects	Lai et al. 2005
ENDOIII/FPG sites (comet)	Policemen from Prague, Czech Republic (65)	M 31 and 35 years (median) NS	PM_2.5_ (stationary monitoring data, 33 ± 40 and 15 ± 9 μg/m^3^) PAHs (personal exposure, 8.5 ± 9 and 3.0 ± 3.4 ng/m^3^)	Exposure	Highest level in WBCs of exposed subjects; positive correlation between PAH exposure and DNA damage in samples collected in January	Novotna et al. 2007^e^
FPG sites (comet)	Subjects exposed to traffic (44) and controls (27)	MF 35–64 years S/NS	None	Exposure	Statistically nonsignificant higher level in WBCs of exposed subjects	Palli et al. 2009
8-oxodG (ELISA) 15-F_2t_-isoprostanes (immunoassay)	Bus drivers (50) and controls (50)	M 50 ± 10 and 51 ± 11 years NS	PM_2.5_ and PM_10_ (stationary monitoring station) and PAHs (personal exposure) PM_2.5_, 32.1 ± 8.1 and 20.9 ± 6.8 μg/m^3^ PM_10_, 38.6 ± 8.2 and 24.1 ± 6.5 μg/m^3^	Biomarker exposure	Highest levels in urine of exposed subjects	Rossner et al. 2007, 2008a, 2008b^f^
8-oxodG (LC-MS/MS) M_1_dG (immunoslot blot)	Policemen, bus drivers, and controls (356)	M 34.1 ± 9 years S/NS	Concentration of PAHs in personal PM_2.5_ samples	Biomarker exposure	Policemen in Kosice, Slovakia, had higher levels of 8-oxodG in WBCs than did controls; no effect in policemen from Prague; 8-oxodG levels were very high (i.e., 53.6 lesions/10^6^ nucleotides, corresponding to 244 lesions/10^6^ dG); significantly higher levels of M_1_dG in exposed subjects in Sofia	Singh et al. 2007

Abbreviations: ECD, electrochemical detection; ELISA, enzyme-linked immunosorbent assay; F, female; LC-MS, liquid chromatography–mass spectrometry; LC-MS/MS, liquid chromatography with tandem mass spectrometry; M, male; MF, male and female; NR, not reported; NS, nonsmoker; S, smoker; SPM, spectrophotometry.

a The values represent exposure assessment in the high-exposure and low-exposure group, respectively, unless stated otherwise by specific footnotes.

b We used data from bus drivers on working days.

c We pooled means and SD from smokers and nonsmokers of controls and traffic officers at the sampling before the intervention with phytochemicals (day 0).

d The publication reports the mean level DNA damage without indication of the SD, whereas later studies by the same group showed a coefficient of variation of 40%.

e The data represent the variation between sampling in January and September. The personal exposure to PAHs in the exposed and control group was 6.0 and 4.5 ng/m^3^, respectively.

f The study had sampling of PM_2.5_ and PM_10_ by personal monitors, but the low amount of material precluded the assessment of individual exposure.

**Table 5:** Summary of cross-sectional studies on exposure to air pollution PM from combustion processes in different areas.

Biomarker	Subjects (*n*)	Sex, age, smoking	Exposure assessment^a^	Potential measurement error	Findings	Study
FPG sites (comet)	Taxi-motor drivers, people living/working near busy roads and rural controls (135)	M 34 ± 10 years NS	Ambient (stationary) sampling of UFPs (201,691 and 6,961 particles/cm^3^) (midday 1-hr concentration in a busy street intersection and town square in a rural village, respectively) and urinary excretion of *S*-PMA	Exposure	Association between S-PMA excretion and FPG sites in WBCs	Avogbe et al. 2005
8-oxodG (HPLC-ECD)	Children living in rural and urban area (75)	M 9–13 years NS	Benzene (ambient monitoring and personal exposure)	Exposure	Increased in WBCs and urine	Buthbumrung et al. 2008
8-oxodG (immunohistochemistry)	Children living in a low-polluted area and Mexico City (98)	MF 6–13 years NR	O_3_ (stationary monitoring data)	Biomarker exposure	Higher level in nasal biopsies from children in Mexico City compared with children in the low polluted area	Calderón-Garcidueñas et al. 1999
8-iso-PGF (ELISA)	Subjects living in areas of high and low pollution (120)	MF 18–22 years NS	PM_10_, 42.3 (25.7–67.9) and 25.6 (17.8–28.6) ppb NO_2_, 39.7 (8.3–49.9) and 21.6 (11.4–29.6 ) ppb O_3_, 42.9 (28.5–65.3) and 26.9 (17.6–33.5) ppb (stationary monitoring stations with subsequent modeling)	Biomarker exposure	Highest level in plasma of subjects living in the most polluted area	Chen et al. 2007^b^
8-oxodG (ELISA)	Subjects living in Flanders, Belgium (399)	MF 50–65 years S/NS	1-HOP (urine) *tt*-MA (urine)	Biomarker exposure	Association between exposure biomarkers (1-HOP and *tt*-MA) and 8-oxodG excretion in urine	De Coster et al. 2008^c^
TBARS (SPM) and CDs (SPM)	Medical doctors who lived in (24) or who recently moved to (21) Mexico City and controls (17)	NR 17–32 years NS	O_3_, 152 and 29 ppb (stationary monitoring)	Biomarker exposure	No difference in serum level between subjects who had permanently or who had never lived in Mexico City; subjects who had recently (within one week) moved to Mexico City had elevated levels in serum	Hicks et al. 1996^d^
TBARS (SPM)	Subjects exposed to residential biomass smoke (28) and controls (15)	F 31–63 years NS	None	Biomarker exposure	Highest level in serum of exposed subjects	Isik et al. 2005
CDs MDA	Children living in Isfahan, Iran (374)	MF 10–18 years NR	PM_10_, 122 ± 34 μg/m^3^ NO_2_, 34 ± 13 ppbO_3_, 38 ± 12 ppb SO_2_, 36 ± 14 ppb (stationary monitoring)	Biomarker exposure	Association between PM_10_ and CDs in plasma	Kelishadi et al. 2009^e^
TBARS (SPM)	Subjects living in rural (125) and urban (167) areas of Mexico	MF 34 ± 6 and 69 ± 8 years NS	O_3_ (155 vs. 46 ppb) PM_10_ (122 vs. 104 μg/m^3^) Stationary monitoring station	Biomarker exposure	Highest level in plasma of subjects living in Mexico City	Sanchez-Rodriguez et al. 2005^f^
8-oxodG (HPLC-ECD)	Subjects living in a rural village (100) and two suburbs of Antwerp, Belgium (100)	MF 17.2 ± 0.8 years S/NS	1-HOP (urine) *tt*-MA (urine)	Exposure	Highest level in urine from exposed subjects; no correlations between exposure markers (1-HOP and *tt*-MA) and 8-oxodG excretion in urine	Staessen et al. 2001^g^
8-oxodG (ELISA)	Children living in areas of low and high air pollution exposure (894)	MF 6–11 years NS	PM_2.5_, 22.7 and 16.8 μg/m^3^ PM_10_, 30.0 and 20.4 μg/m^3^ Stationary monitoring station	Biomarker exposure	Positive association between air pollution exposure and urinary excretion of 8-oxodG in the area with high air pollution (Teplice, Czech Republic); same association statistically nonsignificant in the area with low level of air pollution (Prachatice, Czech Republic)	Svecova et al. 2009
ENDOIII/FPG sites (comet) 8-oxodG (HPLC-ECD)	Subjects living in Copenhagen, Denmark (40)	MF 36.5 (27–46 years) S/NS	Benzene (personal exposure and urinary *S*-PMA excretion)	Exposure	Positive association between urinary *S*-PMA excretion and 8-oxodG (WBCs); no associations with ENDOIII/FPG sites (WBCs) or urinary excretion of 8-oxodG	Sørensen et al. 2003c^h^
TBARS (SPM)	Children living in Pancevo (industrial area) and Kovacica (village) in Serbia (128)	NR 12–15 years NR	None	Biomarker exposure	Highest level in plasma from exposed subjects	Vujovic et al. 2009^i^

Abbreviations: CDs, conjugated dienes; ECD, electrochemical detection; ELISA, enzyme-linked immunosorbent assay; F, female; M, male; MF, male and female; NR, not reported; NS, nonsmoker; SPM, spectrophotometry; iso-PGF_2_, 8-iso-PGF_2_, 8-iso-prostaglandin F_2_; MDA, malondialdehyde; SO_2_, sulfur dioxide.

a The values represent exposure assessment in the high-exposure and low-exposure group, respectively, unless stated otherwise by specific footnotes.

b We used the median and interquartile range as surrogates for the mean and SD.

c We used data from Antwerp, Belgium, and a rural area in the analysis because they had emissions of PAHs, and we estimated the SD from the 95% CI.

d We calculated the mean level of LPO products from TBARS and CDs.

e We used data based on the difference in interquartile range of the exposure (PM_10_) and assuming that the median concentration of exposure (122 μg/m^3^) corresponds to the mean level of MDA (0.7 μM) and CDs (2.5 μM). We calculated the SD from the mean coefficient of variation (11%) of the LPO products.

f We pooled mean and SD from adult and elderly subjects.

g We pooled data from Wilrijk and Hoboken, Belgium, for the analysis and calculated the SD from 95% CI.

h We used the mean and SD in the groups of subjects being either higher or lower than the median urinary excretion of *S*-PMA.

i We estimated the SD from 95% CI.
